# Tissue response to the use of synthetic and biological meshes in implant-based breast surgery: A histomorphological case-series of six patients

**DOI:** 10.1016/j.jpra.2026.07.003

**Published:** 2026-07-07

**Authors:** James Stathakios, Peter F. Koltz

**Affiliations:** a University of Toledo Department of Surgery, 3000 Arlington Ave, Toledo, OH, USA; b Promedica Physicians Plastic and Reconstructive Surgery, 7634 W. Central Ave, Toledo, OH, USA; c pēkoMD, 7634 W. Central Ave, Toledo, OH, USA

**Keywords:** Breast reconstruction, Tissue remodeling, Biocompatible materials, Surgical mesh

## Abstract

Despite the widespread use of mesh in implant-based breast surgery, histologic characterization of mesh-associated tissue responses remains limited. This retrospective case series examines the histologic features of 6 tissue biopsy specimens, which feature explanted mesh from 6 patients who underwent revision breast surgery. Revision procedures occurred between 4 and 36 months after initial implantation of the mesh. All tissue samples underwent objective scoring across predefined histologic categories and descriptive pathologic assessment. Evaluation of the specimens revealed immune cell infiltration predominantly composed of macrophages, an acute inflammatory response and foreign body reaction that diminished progressively over time, and organized deposition of an extracellular collagen matrix without fibrosis of the tissue at later time points. These histologic findings may be helpful considerations in the overall evaluation of mesh performance.

## Background

Surgeon preference for mesh use in implant-based breast surgery is often formed both by subjective experience and objective evidence. However, thorough evaluation of mesh performance also requires an understanding of the histomorphologic tissue response to the mesh product. Breast meshes are intended to facilitate tissue remodeling and stabilize implant position while limiting maladaptive responses such as chronic inflammation, foreign body reaction, and fibrosis that contribute to complications including asymmetry, implant malposition or exposure, and capsular contracture.

A recent histologic analysis of breast tissue biopsy specimens containing a poly-4-hydroxybutyrate (P4HB) biosynthetic mesh demonstrated progressive mononuclear cell infiltration, gradual mesh degradation, and organized collagen deposition without evidence of a significant inflammatory response.^1^ Similar patterns of tissue remodeling have been reported in studies evaluating acellular dermal matrices.^2^ Though not included in the present series, it is possible that P4HB mesh may share behaviors with other synthetic absorbable meshes. However, histologic characterization specific to synthetic absorbable meshes used in breast surgery remains limited, and comparative data evaluating tissue responses across synthetic and biologic mesh types are lacking.

In this brief retrospective, single-surgeon case-series, we present histomorphologic evaluation of synthetic and biologic meshes in breast surgery. We characterize the key histologic features of 6 specimens that were collected from 6 unique patients during revision procedures.

## Methods

All patients represented in the sample underwent primary implant-based breast surgery with placement of mesh at a single institution from 2020 to 2024. All index operations featured pre-pectoral implant or tissue expander placement with complete mesh wrapping. Specimens were explanted during revision surgery and included native tissue and mesh. Each patient provided one specimen. The sample included 5 specimens with synthetic mesh (4 TIGR®, 1 DuraSorb®) and 1 with biologic (Cortiva®). Revision procedures were implant exchange in 5 patients and tissue expander-to-implant exchange in one. Ethical approval for this study was obtained from the Promedica Hospital Institutional Review Board. All patients provided written informed consent for surgery and tissue sampling.

The histomorphologic appearance of each specimen was assessed by a board-certified pathologist using two approaches. First, specimens were graded across predetermined histologic categories on a 9-point ordinal scale (from 0–4) at intervals of 0.5. Numeric integer values correlated to the descriptors “absent”, “slight”, “moderate”, “marked”, and “severe”. A score of 2.5, for example, indicated that the histologic changes for that category were somewhere between “moderate” and “marked”. Second, descriptive pathology reports were provided for Hematoxylin and Eosin (H&E) stained slides.

## Results

**Fig. 1:**
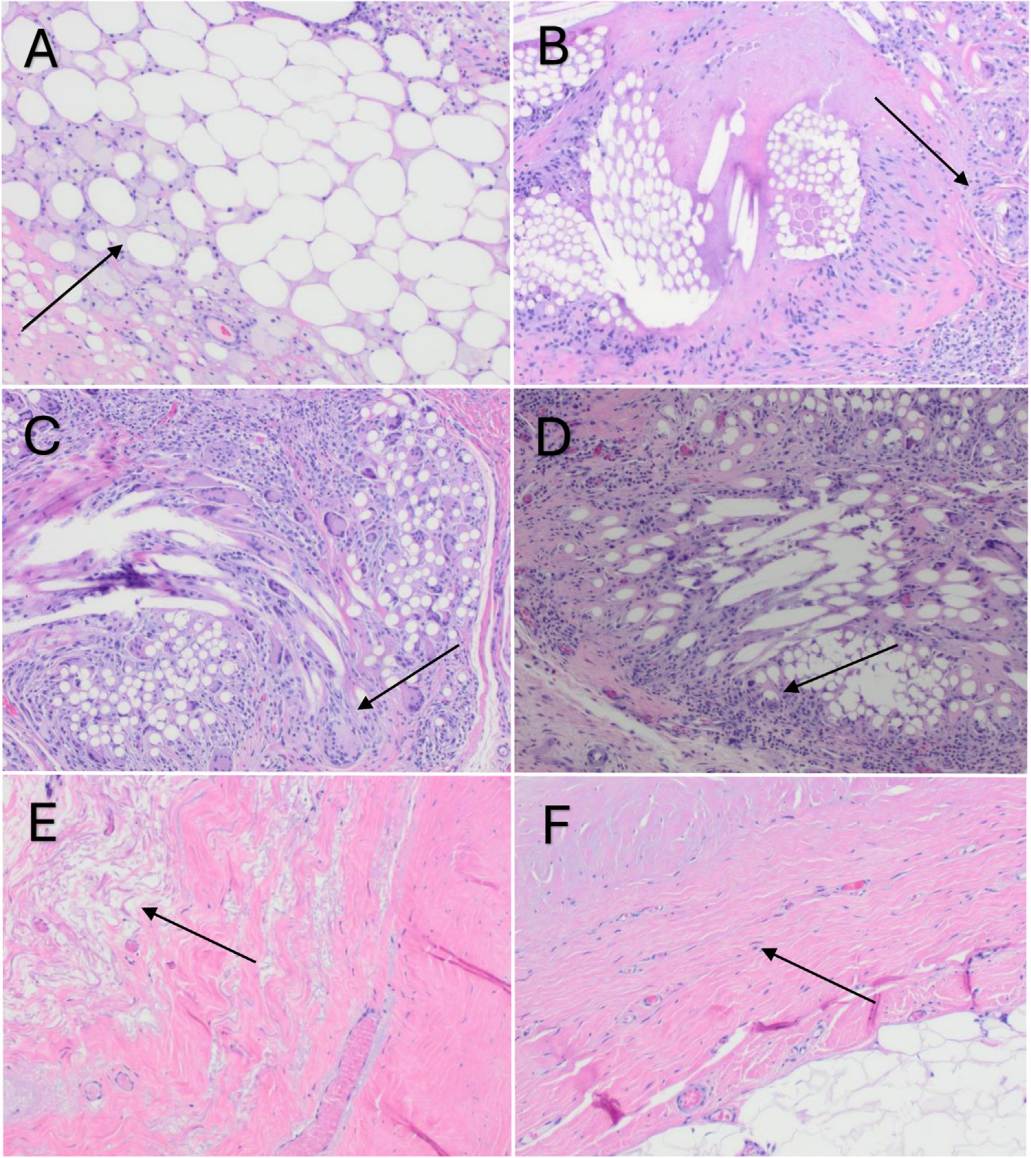
A-F. Representative images of H&E stained slides for each specimen. (A). Specimen 1: TIGR® mesh, explanted 4 months after index operation. Marked neutrophilic infiltrate and moderate chronic inflammatory response including lymphocytes, plasma cells, and abundant macrophages. Moderate giant cell reaction around the mesh product. Arrow: Inflammatory response with fatty infiltration. (B). Specimen 2: TIGR® mesh, explanted 9 months after index operation. Mild neutrophilic infiltrate, slight chronic inflammatory response including lymphocytes and macrophages. Arrow: Marked giant cell reaction predominantly around the mesh product. (C). Specimen 3: TIGR® mesh, explanted 12 months after index operation. No neutrophilic infiltrate, slight chronic inflammatory response including lymphocytes and moderate macrophages. Arrow: Marked giant cell reaction around the mesh product. (D). Specimen 4: TIGR® mesh, explanted 19 months after index operation. Slight chronic inflammatory response including lymphocytes, plasma cells, macrophages. Arrow: Slight giant cell reaction around the mesh product. (E). Specimen 5: Cortiva® mesh, explanted 35 months after index operation. No neutrophilic infiltrate, chronic inflammatory response, or macrophages. No giant cell reaction. Arrow: 1 mm layer of elastin underlying the collagen capsule layer. (F). Specimen 6: DuraSorb® mesh, explanted 36 months after index operation. No neutrophilic infiltrate, no chronic inflammatory response. Few macrophages are present with no giant cell reaction and no mesh product reaction seen. Arrow: Fibrosis and collagen with no inflammatory response.

Median time to explant ranged from 4–36 months after index surgery, with a median of 15.5 months. Review of explanted specimens revealed immune cell infiltration predominantly composed of macrophages, an acute inflammatory response and foreign body reaction that diminished progressively over time, and organized extracellular collagen matrix deposition without fibrosis of the tissue at later time points. A summary of results for each specimen is included in Table 1. Representative images of H&E slides at 10x magnification with accompanying pathology reports are provided in Fig. 1.

**Table 1:** Specimen characteristics and histologic scores.

Specimen ID No	Commercial Mesh Product Used at Index Operation	Index Operation Implant Characteristics (Type, texture, material, volume)	Time to Explant (Months)	Neutrophils	Macrophages	Giant Cells	Fibroblasts	Thickness of Collagen
1	TIGR®	AlloX2 tissue expander, smooth, silicone, filled to 460 mL	4	2	3.5	1.5	2	2
2	TIGR®	Sientra implant, smooth, silicone, 695 mL	9	1	3	3	1.5	3
3	TIGR®	Sientra implant, smooth, silicone, 255 mL	12	0	3	3	1.5	2.5
4	TIGR®	Sientra implant, smooth, silicone, 330 mL	19	0	2	2	1	1.5
5	Cortiva®	Sientra implant, smooth, silicone, 440 mL	35	0	0.5	0	0.5	2.5
6	DuraSorb®	Sientra implant, smooth, silicone, 600 mL	36	0	1	0	1	1

Scores are presented as an ordinal scale with numeric values between 0–4 at intervals of 0.5. Integer values, beginning with 0, correlate to the descriptors “absent”, “slight”, “moderate”, “marked”, and “severe”.

## Discussion

Across all samples, macrophages were the most frequently observed cell-type. Neutrophils were present only in the 2 specimens with the shortest explant intervals (4 and 9 months post-implantation). H&E slides for these specimens demonstrated neutrophilic infiltrate, mixed inflammatory changes, abundant macrophages, and a foreign body giant cell reaction surrounding the mesh product (Fig. 1A and B). These findings are consistent with the expected initial inflammation that occurs prior to the proliferative and remodeling phases of wound healing.

All specimens containing TIGR® mesh demonstrated multinucleated giant cells. Given its biphasic degradation profile with complete resorption occurring over 3 years,^3^ the presence of residual mesh material at the time of explant (4–19 months for TIGR® specimens) likely accounts for the observed continued foreign body reaction. In contrast, Cortiva® biologic mesh is typically integrated in the surrounding tissue over 3–6 months,^4^ while DuraSorb® synthetic mesh undergoes complete resorption by 9 months.^5^ The specimens featuring Cortiva® and Durasorb® mesh (which were explanted at 35 and 36 months, respectively) showed no residual mesh product, no giant cells, and no chronic inflammatory response.

As wound healing progresses to its later phases, fibroblast activation leads to collagen deposition and extracellular matrix organization. Prolonged inflammatory activity may result in fibroblast overactivation, excessive collagen deposition, and fibrosis, which contributes to complications such as asymmetry, implant malposition, and capsular contracture. Across our sample, collagen deposition was organized without evidence of tissue fibrosis. Capsule thickness was not scored as “severe” in any specimen, suggesting no significant fibrotic changes in our sample.

## Limitations

This study has several important limitations that must be considered. Most notably, generalizability is limited by the small sample size, variable explant intervals, and single surgeon experience. Additionally, the presence of an implant may independently influence tissue response at the implant-mesh interface. Similarly, the underlying reason for revision may represent a confounding variable which affects the histologic findings. Therefore, this case-series is not intended to demonstrate causality, but rather to serve as a foundation for future studies that evaluate mesh products and tissue responses.

## Conclusion

Our study is among the first to provide histologic evaluation of synthetic mesh in breast surgery, and to directly compare tissue responses across mesh types. When coupled with clinical outcomes data and individual patient considerations, these histologic findings may be helpful for surgeons when selecting which mesh product to use in implant-based breast surgery.

## Funding statement

This research did not receive any specific grant from funding agencies in the public, commercial, or not-for-profit sectors.

## Ethical approval

Ethical approval for this study was obtained from the Promedica Hospital Institutional Review Board. All patients provided written informed consent for surgery and tissue sampling.

## Declaration of competing interest

The authors have no conflicts of interest to disclose.
